# Improving the installation of renewable heating technology in UK social housing properties through user centred design

**DOI:** 10.1177/1420326X15598819

**Published:** 2015-11

**Authors:** Natalie Moore, Victoria Haines, Debra Lilley

**Affiliations:** Loughborough Design School, Loughborough University, Loughborough, UK; University College London, UK; University College London, UK

**Keywords:** Heat pumps, Social housing, User centred design, Service delivery, Energy

## Abstract

Social housing organisations are increasingly installing renewable energy technologies, particularly for the provision of heating and hot water. To meet carbon reduction targets, uptake and installation must allow occupants to use the technology effectively. This paper describes research which investigated the service of installing heat pumps into UK social housing properties, from both landlords’ and tenants’ experiences. Adopting a user centred design approach, the research was in three phases: an exploration study to investigate landlords’ and tenants’ experiences of heat pump installation and use; refinement and development of the requirements for improved service delivery, primarily technology introduction and control; and the development and initial evaluation of an information leaflet as a key touchpoint in the service delivery. Recommendations for improved service delivery, to enable heat pumps to be accepted and used more effectively, are presented, as well as reflection on the process of applying user centred design in this context. In a relatively immature area of industry, installations to date have been heavily focused on technical aspects. This paper provides an insight into the human aspects of the service delivery of heat pumps in social housing, providing designers and social housing landlords with insight about how to improve the service.

## Introduction

The domestic sector accounts for around 27% of all UK CO_2_ emissions^1–3^ and is considered very cost-effective to treat in terms of carbon abatement.^4^ Installations of renewable heating technologies are increasing across the UK domestic sector and, as a result of government supported funding, largely in social housing. However, these technologies need to be operated effectively to achieve optimal energy efficiency, both in terms of energy consumption and operating costs for the householder. Retrofit measures may only be half as effective as expected if not installed and monitored adequately or due to subsequent inefficient use of heating.^5^

This paper describes the research that adopted a user centred design approach with the aim to define requirements of specific stakeholders in the social housing system when introducing heat pumps and to identify ways of delivering a better service with a view to improving levels of uptake and efficiency of use. It attempts to answer the following questions: 1. What lessons can be learnt from previous experiences when retrofitting heat pumps technologies into social housing? 2. What measures would help augment the successful introduction and adoption of heat pumps in the future?

This research was completed as part of the RCUK/E.ON funded research project “Carbon, Control and Comfort: user centred control systems for comfort, carbon saving and energy management” (Grant number EP/G000395/1). The research aimed to devise interventions that would assist UK social housing tenants to reduce domestic carbon emissions from their heating practices, whilst enabling satisfactory levels of comfort to be maintained. A key component of the research was user involvement, in order to define and address, in context, how renewable heating technology might fit into the lives of the users. User involvement is a widely accepted principle in the development of usable systems^6–8^ and the earlier it occurs, the fewer potentially costly modifications are required to make something usable in retrospect.

### Social housing and energy efficiency

Social housing, comprising 18% of the domestic stock (4.7 million homes) in the UK,^9^ provides rented accommodation below market price for people on lower incomes.^10^ It is often referred to as affordable housing, as it provides secure, decent housing for people who do not have the means to buy a property or rent a home in the private market.^11^ This sector of housing is owned and managed by local authorities (councils) or registered social landlords, which are either housing associations or Arm’s Length Management Organisations.

Social housing organisations are service providers.^12^ In managing social housing properties, landlords provide many different services to tenants, such as property maintenance and consumer advice. Installing a heat pump system into a social housing property is a service delivered by the social housing provider to the tenant. It is not just the handing-over of a product; there are surrounding service elements such as tenant consultation, assistance with usage and on-going maintenance of the product which all impact on the usability of the system by the tenant. The experience of social housing tenants throughout the service delivered by the social housing landlord may ultimately have implications for their willingness to accept the new heating system, use the system correctly and their likelihood to recommend it to other tenants.

Although the way in which a social landlord works with and for their tenants can vary in approach, it has been found that there are some commonalities across social housing organisations in the UK: a paternalistic relationship between landlord and tenant, a lack of choice over accommodation and rising tenant expectations.^13^ With limited choice for the tenant over the location and type of property they receive, alongside their increasing expectations, providing choice over what services they receive (and how) is becoming even more important to ensure tenant satisfaction.^13^ This has implications for the level and type of engagement with tenants to ascertain their needs. This engagement of tenants is crucial to social housing landlords, as their performance is measured by regulators, by tenant interaction and satisfaction.

Research indicates that a key barrier to the implementation of renewable technology into properties is the upfront capital cost of the installation but in the social housing sector, upfront costs are the social landlord’s responsibility.^14^ Whilst social housing tenants have less choice over their homes than private homeowners, they do need to accept the new renewable technology and run it efficiently for the expected savings to be made. Ground source heat pumps and air-to-water air source heat pumps use a wet central heating distribution system and deliver lower temperatures for a longer period of time. To work efficiently, under floor heating or larger radiators are more suitable, providing a larger surface area to distribute heat. Compared to conventional boiler systems, the temperature emitted is lower, which provides a different thermal experience to the householder. However, heat pump technology is important as it potentially offers significant carbon saving opportunities for future.^15^ However, some research suggests that technologies such as heat pumps are viewed as less effective than more established technologies.^9^

Much of the research to date has concerned heat pump performance purely from a technical perspective. Penwith Housing Association in Cornwall, UK, completed the first project to retrofit ground source heat pumps into existing social housing in 2004. They reported that tenants were happy with the new systems and that results showed ground source heat pumps can provide space and hot water heating at an affordable cost.^16^ They also stated that one of the most significant outcomes of the project was the encouragement to other social housing landlords to install ground source heat pumps.^16^ The Energy Saving Trust conducted the first large-scale field study of UK heat pump installations that included user experience. The study monitored technical performance as well as customer behaviour, with the aim of informing industry stakeholders of improvements that could be made to installations.^17^ This project however incorporated a diverse sample, including privately owned properties and social housing dwellings, installations in new-build properties and retrofitted into existing properties. The findings suggest that customer behaviour is a variable that impacts performance. The report also states that many householders had difficulties understanding the instructions for operating their heat pump, and called for clearer and simpler customer advice.^17^ BRE also found a lack of understanding amongst tenants about how their heating system worked and that providing better information could address this.^18^

Grant funding has given social housing organisations the opportunity for numerous installations and, therefore, heat pump installations have been increasing in social housing. However, getting the technology right and making it affordable is still not always enough to get new technologies adopted or used as intended.^19^ As heat pumps are delivered as a service from the social housing provider to the tenant, considering the design of the service therefore becomes imperative to achieve successful uptake and effective usage.

Service design is an emerging discipline^20^ and becoming established in industry and academia.^21^ It is recognised as an interdisciplinary approach, focusing on the design of interactions between users and the supply system that form a service.^22^ Although services themselves are intangible, service design addresses the ‘service interface’ – also referred to as ‘service encounters’ or ‘touchpoints’ – which encompasses the tangible or visible parts of the service which support the user experience and result in its existence and delivery.^22^ As a developing discipline, there is no single, refined definition of service design. The UK Design Council’s definition states that “Service design is all about making the service you deliver useful, usable, efficient, effective and desirable”.^23^ However, the principles of its process are clear: that it is user centred, that it is co-creative, involving users throughout the process and giving them the opportunity to participate in the design stage, producing design ideas themselves. Whilst service design is evidently a user centred approach in its philosophy and methodology, the second core principle of it being a co-creative process is what differentiates the service design methodology from the traditional user centred design approach. In some contexts, co-creation is not possible. In the social housing context, the residents may be difficult to engage in heavily participative, co-creation methods, and so a user centred design approach was taken, to focus on the users’ requirements without relying on significant design contribution from participants.

The principal ideals of user centred design are to create the best possible outcome for the end user by understanding their requirements and designing what attends to those needs.^24^ Obtaining knowledge of the users and developing interventions with a user focus are achieved by involving the user in the design process. User centred design advocates the involvement of users from the earliest stage, to understand them and the context, and maintain a focus on them to deliver suitable and beneficial end products or systems.^25^ Applying user centred design to a service is somewhat different, however, to applying it to a product or interactive system, as is its traditional application. Services are complex systems involving a multitude of interactions of various people and objects over time.^12^ The user centred design process can be applied to the service context but this level of complexity impacts the approach. Instead of just identifying the end user of a product or system, wider service actors must be considered and so a range of service recipients end and service providers was involved in this research programme.

## Method

Adopting a user centred design approach, three main phases to the research were undertaken. A first exploration study used semi-structured interviews to understand the context surrounding heat pump installation, investigating the experiences of installation and use from both landlords (*n* = 7) and tenants (*n* = 34) drawn from six English social housing organisations. Through this understanding, the needs and wants of both landlords, as the service provider, and tenants, as the service recipient, emerged, with potential areas to be addressed to improve the service identified. A second study was undertaken with stakeholders on both sides of the service provision from one particular local authority, through a combination of focus groups and interviews (*n* = 14 service recipients and *n* = 8 service providers). This continued the user centred design approach, developing user requirements and ascertaining ways in which these key requirements could be met. A particular measure, in the form of an information leaflet, was prototyped, based on the requirements extracted from the first exploration study and further developed in the second. This intervention was then sent to a selection of social housing tenants and their opinions on it gathered via a questionnaire (*n* = 15). This applied the final stage of the user centred design process (evaluation) to a key touchpoint of the service delivery.

**Figure 1.**
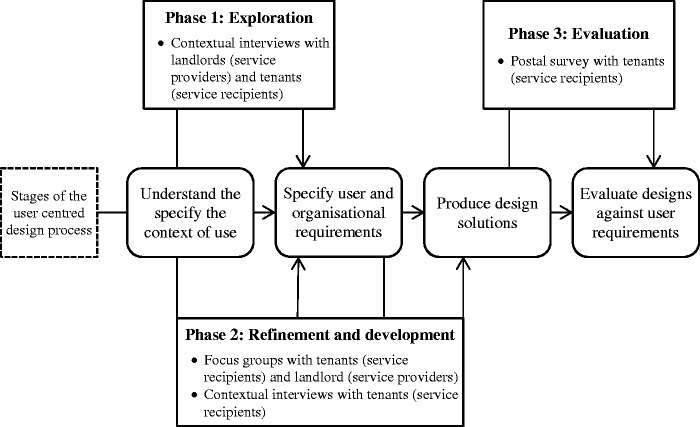
Research methodology for the user centred design approach.

The relationship between the various studies described in this paper and the user centred design process^26^ is shown in Figure 1.

### Phase 1: Exploration

The existing literature has indicated that people’s understanding of heat pumps is limited and that the information provided could be improved. The exploration study enabled a more detailed investigation to explore these issues further and identify any other positive or negative experiences with heat pumps in social housing, identifying where improvements could be made to the installation service. The exploration study comprised of interviews with employees from social housing organisations and social housing tenants, through interviews in the home or by telephone. As participants were difficult to recruit, an opportunistic sample of participants that met the criteria was used; this included social housing landlords who had been involved in the installation of heat pumps in their properties (*n* = 7) and social housing tenants who had a property with a heat pump (*n* = 34) from six geographical locations across England.

Interviews with participants lasted up to 1 h and were conducted in their homes (tenants) and workplaces (landlords) or, where this was not possible, by telephone. Interviews were audio-recorded and later transcribed. Thematic analysis^27^ was used to identify insights and requirements in the service process as well as any positive and negative elements of the heat pump installation process. This highlighted potential areas for improving the service.

### Phase 2: Refinement and development of requirements

The second phase of the research refined and developed the user requirements identified in Phase 1, exploring not just what needed to be improved but how this could be done. The purpose of the study was to form a deeper contextual understanding, generate ideas for potential improvements to aspects of communication in the installation service and to obtain insights and user-generated ideas for requirements of how these should be developed. One of the social housing organisations that participated in the first exploratory phase continued their participation in this phase, which resulted in the identification of requirements for a physical touchpoint – an information leaflet.

The service providers (landlord representatives and installers, *n* = 8) took part in a 2-h focus group. Both landlord representatives (Energy Officers, Contract and Business Support Offices) and installers were included in the study, as it was identified in Phase 1 that they both form key communication channels, interacting with the tenants directly. The focus groups built on the findings of the first Phase to focus on lessons learned through existing installations and areas for improvement.

All tenant participants were heat pump users to provide substantial, relevant information, based on their experience.^28^ Tenants with heat pumps (*n* = 14) were recruited to take part in a 90-min focus group or individual contextual interview, where it was not possible for them to travel to the group discussion. A modified interview schedule was developed for these interviews, aiming to elicit information comparable to the group data collection and to validate and develop further the findings. In the group discussions, participants’ experiences with the heat pumps were discussed; participants were asked to make suggestions about how they thought their experience or service could have been improved. After a break, participants focused on heat pump control. They were asked about their experience of controlling the heat pump and the control devices themselves and what they felt could be done to improve this element of the system. Lastly, the participants discussed what they would like in terms of information provision.

All interviews and focus groups were audio-recorded, transcribed and analysed using thematic analysis.^27^ Specific requirements for improved information were highlighted and so the final phase of the research involved development and evaluation of a prototype information leaflet.

### Phase 3: Evaluation

Based on the requirements identified in Phase 2, two sets of leaflets were produced: one system-specific (ground-source or air-source) leaflet to be given when the tenant is approached about having a heat pump installed (leaflet A) and a system-specific leaflet for tenants at the time of installation (leaflet B).

The evaluation of the information leaflets was undertaken by social housing tenants (*n* = 15) from the same housing association used in Phase 2, but involved new participants, all of whom had heat pumps. Although the evaluation could be done by residents without a heat pump, they would have needed to have been provided with significant accompanying guidance to clarify contextual issues. As their experiences of heat pumps would have been very limited, they were not approached for this phase of the research. The study aimed to test the usability of the heat pump information materials and identify whether they met user requirements by obtaining users’ opinions on the design and content of the information materials. A postal questionnaire survey was selected as an appropriate method for this study design, to provide independent views, completed at the participants’ convenience, within a specified timeframe. The questionnaire was designed to explore both leaflets A and B, including questions to obtain opinions about the content and aesthetic presentation. Participants could write or draw on the prototypes if they felt it would help them clarify any responses. The tenants were requested to response within three weeks and all surveys received by that deadline were included in the analysis. Following piloting, 120 surveys were distributed by the social housing landlord to households known to have a heat pump, with the household receiving either leaflet A or leaflet B, randomly assigned, but relevant to their particular system (ground-source or air-source). Those who wished to take part completed the questionnaire survey and returned it with the annotated leaflet prototype in the provided stamped, addressed envelope directly to the researchers.

## Findings from Phase 1: Exploration

### Exploratory themes emerging from landlords (service providers)

The initial decision for all landlords of where to install heat pumps was determined primarily by the property, not the occupants. However, landlords all said that their tenants had a choice about whether to have a heat pump installed or not. Tenants were first approached in writing, then visiting them at home; some landlords ran community information events for tenants to attend together to find out more about the heat pump systems. According to the landlords, the tenants had the option to refuse the installation and in some cases there were refusals, despite further attempts to engage the tenant. The majority of landlords interviewed ran heat pump installation programmes on a community scale to impact multiple properties at one time. In all cases, however, installation programmes for retrofitting heat pumps were entirely dependent on funding being available.

A number of key themes relating to heat pump installation emerged from the landlord interview data. These are shown in Table 1 in priority order of importance, determined by the frequency of the issue being mentioned or the implied importance.

**Table 1.** Issues raised by the landlords during the exploration study.

Emerging theme	Detail of issues raised
Information provision	Tenants told not to touch controls
	Written information inconsistent
	Written information not clear
	Information needs to set appropriate expectations
Introducing heat pump technology to tenants	Initial approach was usually by letter
	Home visits frequent
	Introducing the technology was ‘selling the technology’
	Requests for repeat visits
Effect of community	Neighbours influence promotion of heat pumps
	Neighbours influence usage of the heat pump
	Community information events were beneficial
Selection and impact of contractors/workmen	Standard of service important
	Location of maintenance teams sometimes too far away to be effective
	Tenant contact
Tenant choice	All tenants had a choice about having a heat pump
	Refusals were experienced
	Some barriers to adoption
	Tenants referred to as customers
Effect of tenant’s age on installation	Age did not affect service delivery
	Older tenants were a target population for heat pump installations
Reasons for installation	Property-driven: off-gas properties
	Benefits over other heating system options
	Fuel poverty alleviation
	Legislation/obligations
	Tenant need/request
Funding	Necessity to enable installations
	Determinant for amount and timing of installations
Type of system installed	Varied opinion on the systems available
	Property and cost drive the choice of technology

Landlords reported that they were conscious that the level of understanding by tenants was varied and often had to verbally communicate to tenants how the heat pump worked. One participant commented:

“A lot of the teething problems were about how to use the actual systems and that’s more about education of the tenants … They’re very used to having a physical fire or physically turning the heating up to feel warmer whereas it’s been a very different educational process with them to try to get them to understand.” (Landlord LE)

However, there was inconsistency over the written information provided by the different landlords. Some housing associations appeared to deliver more substantial written information when introducing the heat pump technology to tenants, for example:

“We did them an information literature leaflet that told them all about the process of the drilling, the process of what the heat pump was, how the heat pump worked, how it would heat their home and what the expectations from the heat pump would be.” (Landlord LC)

Landlord LA reported that some tenants looked at heat pumps on the Internet to help inform their decision about whether to have a heat pump. Landlords were also aware that tenants talked to each other about their heat pump systems, recognising the community benefits of social housing. Some landlords reported that tenants visited other properties with a heat pump installed, to see contextually what the installation was like in a home.

Landlords reported that problems occurred when tenants tried to alter the settings, without understanding how to do so correctly. It was common amongst the interviewed landlords that they promoted setting the heat pumps themselves and told tenants not to touch the controls. However, written information to help tenants with usage of the system was consistently highlighted as an area for improvement by landlords.

The landlords referred to tenants as customers, in a manner that portrays the relationship between them as a service provider and service recipient. When the landlords talked about approaching tenants about having the heat pump they spoke in terms of ‘selling the technology’.

### Exploratory themes emerging from tenants (service recipients)

The interviews with tenants provided insights relating to their opinion, understanding and usage of the heat pump system and their positive and negative experiences of the service. These are presented in order of importance as mentioned in Table 2.

**Table 2.** Tenant exploration study findings.

Emerging theme	Detail of issues raised
Information provision pre-installation	Verbal information could be mixed
	Lack of helpful written information
	Mixed messages communicated
Understanding of the system in use	Radiators not very hot
Control of the system in use	Wariness of using controls
	Understanding of controls
	Use of thermostats positive
	Feelings of vulnerability
Introduction to the system	Initial introduction by letter good
	Home visits by landlord appreciated
Tenant choice pre-installation	Perception of choice mixed
	Barriers to adoption
Disruption during installation	Low level of dissatisfaction
	Unexpected problems dealt with
Impact of contractors	Customer service in the home
	Communication with tenants mixed
Overall satisfaction	Mostly dependent on warmth in the home and cost of the heating
Community effect	Communication between tenants positive
Impact of previous system	Better satisfaction compared to previous systems
Use of technology	Varied technology use
Warmth preferences	Varied temperature settings
	Individual differences
Use of windows	Keeping windows open
Use of additional artefacts for warmth	Use of the electric fire
	Use of a blankets and clothing

There were evidently different stages at which tenants were provided with information about the heat pump system, sometimes when the heat pump technology was introduced to them initially, and sometimes not until the installation process itself. Some tenants, depending on social housing provider, received written information when they were first approached about having a heat pump installed; however, the majority of those interviewed only received verbal information. Some tenants also repeatedly reported not being given any information about how to operate the heat pump, which clearly caused negative feelings amongst the tenants, for example:

“I said to them is there any leaflet or a booklet on this to… how to look after it or adjust it? Oh no. We don’t give you them, he said. He said people mess about and they all go wrong. So he said we don’t give you them… I asked for a book… a leaflet to tell me how it worked…But I never got one.” (Tenant 6bM)

Other tenants were able to get key information from the installer, to assist with their on-going operation of the heat pump:

“He [maintenance] wrote them [control instructions] out and I stuck them up on the heat pump.” (Tenant 5bF)

All tenants who had a thermostat control device reported being able to use it, although it was evident from their comments that this was limited to simply changing the temperature rather than programming the heat pump. Tenants consistently reported that they were wary of using the controls and were dependent on the landlord to operate the system. Others reported that their neighbours operated the heat pump controls for them, if the neighbour understood better or had more confidence in using the system. This highlights an issue of vulnerability as a consequence; one tenant reported that her inability to control of her heat pump resulted in her having no heating or water on occasions, until the landlord arrived to assist. There were also reports of opening windows and doors to regulate the internal temperature, with the obvious waste of energy, as tenants were unable to regulate the heating when too hot.

There was a mixture of understanding regarding the heat emitted from the system and there were frequent comments that the radiators were not very hot. Tenants used supplementary heating to provide additional warmth, or used blankets and clothing. Some tenants understood that they had larger radiators because the heat pump ran at a lower temperature; others reported that they thought it was an issue that the radiators were not very hot. Some tenants identified that the radiator temperature being lower meant that they were safer. The responses from tenants appeared to reflect information that they were provided by either the landlord or installers, suggesting that information was retained.

Tenants consistently reported being told that having a heat pump installed would be cheaper than their current system and save them money and this was the key driver for their decision to have it installed. Other reasons included the environmental benefit, cleanliness of the heat pump system or a lack of choice because their current heating system was no longer functional. However, a level of dissatisfaction was exhibited by tenants who claimed that the information they were given when they were first approached was inaccurate after experiencing having the heat pump installed. Tenants also explained they were written to by the landlord to say they were being offered a heat pump. Home visits and personal explanations generally helped the tenant make a decision about the technology and its use. Some responses from tenants indicated however that it did not completely feel like a choice; for some it felt it was inevitable.

Tenants repeatedly reported that they communicated with their neighbours about the heat pump systems, reflecting the comments made by the landlords. They either spoke to each other about having it installed in the first place or how to use it. Community information events appeared to be a successful approach for landlords to inform tenants about the heat pump systems and for tenants to discuss issues amongst themselves.

It was evident from the tenant interviews that the contracted workmen played a significant role in the service delivery. They were the people in the tenants’ home daily carrying out the work and in regular contact with the tenants and so play an important part in the service delivery. Tenants were satisfied when workmen took care in their properties during the installation, covering flooring and furniture and cleaning homes and gardens thoroughly afterwards. The majority of tenants stated they had no problems living at home during this time, especially if they developed a good rapport with the workmen. Some tenants reported more difficulties living at home whilst the heat pump was installed when the installation was carried out during winter months, as they had no heating during this time; tenants also reported it taking a few days to fully heat the fabric of the property once the heat pump had been installed.

### Service actors and touchpoints identified from the exploratory Phase 1

**Figure 2.**
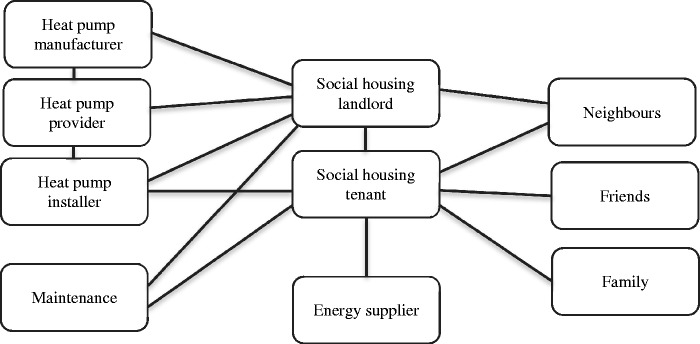
Service actors map.

Participants in Phase 1 often mentioned different actors involved in the service provision; these are shown in Figure 2. The lines signify where there is direct communication between the actors.

The manufacturer, provider, installation contractors and maintenance actors may all be part of the same organisation but each role is identified separately as their interaction with the tenant is different. The study highlighted the significant importance of the heat pump installer in the system through their communication with tenants, therefore they were actively involved in the second phase of this research.

An understanding of the service process was also developed. Table 3 shows the three key stages of the installation service and identifies the interactions (or touchpoints) between the service providers and service recipients.

**Table 3.** Service touchpoints identified through the exploration study.

Introduction	Installation	Usage and maintenance
• Initial letter from landlord	• Home visits from landlord	• Home visits from contracted or in-house maintenance teams
• Home visit from landlord	• Installation work carried out in the home by installers	• Tenants telephone landlord with any queries or problems
• Survey at home by installer	• Verbal information from landlord and installer	• In some cases, tenants contact the contractors (installer or maintenance) directly with queries of problems
• Verbal information from landlord and installer	• Written information about the heat pump system (not in all cases)	• Tenants discuss usage with neighbours, friends and family
• Written information about the heat pump system (not in all cases)	• Tenants telephone or speak in person with landlord about queries or problems	
• Community information event held by landlord (not in all cases)		

The key user requirement derived from this exploratory study was for improved written information to be provided to support the verbal communication from landlords and installers. Inconsistent information provision emerged as the most important issue to address, as it affected tenants’ ability to understand and control their heat pump systems, putting more pressure on the service providers to compensate. Information materials were therefore the priority focus of the continuing research.

## Findings from Phase 2: Refinement and development

### Refinement and development of themes emerging from landlords and installers (service providers)

As established in Phase 1, the installers play a key role in the service provision and tenant experience. The installers reported that they give tenants information on what to expect during the installation as well as how to use the heat pump, including advice on home behaviours regarding the heating, at the initial site inspection of the property. There is also regular contact between the social housing organisation and the installers; they have a good relationship which aids the process, according to both sides. Landlords and installers agreed that the major problem with heat pump installations is the tenants’ poor understanding of the heat pump system and the design of the controls themselves. All landlord and installer participants thought that heat pump controllers are too complicated for users and certainly more complex than necessary. They felt that the control functions could be prioritised and only the necessary functions should be on the control device. The landlords and installers discussed the benefits of standardising the control devices across installations, to make the information provision and internal management easier for frontline staff. This would streamline the process as one of the major problems currently is the diversity of systems and control devices and not knowing what is installed in which property.

In response to questions about setting expectations for monetary savings, the participants stated that caution must to be applied as these savings are not always realised following installation of the heat pump and so tenants can become very dissatisfied. Landlords stated that the running cost information is difficult to predict with changing energy prices, suggesting that giving information about savings in kWh would be more appropriate; however, it was recognised that this would be less understandable by tenants than if delivered in monetary terms.

### Refinement and development of themes emerging from tenants (service recipients)

All of the tenants in the Phase 2 study reported that they received insufficient or inadequate information on how to use the systems. Some felt they were given helpful verbal information when it was installed, but that they required more detailed, specific information to refer to during usage. They require some written information, which is easy to understand, to explain the controls and symbols on their control device. Participants felt that council staff should have better training with regard to heat pump systems so that they could be confident when talking to tenants about their system.

There was a mixture of opinion from participants over whether they had been prepared enough, prior to installation, for what would happen during the installation. Tenants stated they were told about the heat pump system and how it worked. However, the tenants with ground source heat pumps reported that they were not prepared for the size and placement of the internal heat pump unit; they did not expect to lose the substantial storage space, such as airing cupboards, when the units were installed inside the home.

The majority of the participants were happy with the service they received at the installation stage, and were satisfied with the standard of service carried out by the contracted workmen. There were some reports of some impolite and disrespectful behaviour from workmen in terms of handling their personal belongings in the home, but, in these cases, the housing organisation was alerted to this fact and dealt with the problem effectively. Most participants said they would have benefited from a follow-up from the landlord or installer to check how they were getting on with the system, whether they were running it suitably and to answer any questions. They felt this would be especially helpful a few weeks after the system was installed, to allow a period of adjustment. There was a strong opinion amongst the participants that this should be a personal visit and that a telephone call would not suffice; maintenance engineers carrying out checks could see the tenant at the same time to make it an easier task.

There were specific recommendations from participants about the format of improved written information. This should include information on:

- What to expect practically during installation of the heat pump?
- What to expect practically during usage?
- Explanations of functions and symbols on their heating controls.
- How to turn the heating on and off?
- How to change the temperature?
- How to set the programme timer?
- Expected running costs.
- What to do in an emergency?
- Contact information for more help.
- Guidance on what electricity tariff is the most suitable and cost-effective.

The information should be simple and clear, in ‘plain English’ so it is easy to understand. Participants also emphasised that text size and font should be considered for older people with poorer eyesight. It was felt that some of the existing manuals were poorly organised, causing the reader to constantly change between sections pages when trying to read an instruction. The general consensus across all participants was that written information should be presented as a short leaflet with pictures and labels to help explain functions and symbols. In addition to this, information on the best ways to ‘live’ with a heat pump were requested; most tenants commented on manipulating the heating by opening or closing doors and none were certain that they were doing the correct thing.

### Refinement of requirements

Clearly there is a mismatch between what was provided to the tenants about their heat pumps and what they wanted, evidenced by the preference to phone up whenever help was needed, rather than rely on the information already given. Study 2 identified a set of more specific user requirements for the written materials which informed the development of the prototype heat pump information materials. This included a shorter document than is currently provided, written in simple understandable language. Pictures and labels are needed to help explain functions and symbols and the document needs to be sized to fit in the existing tenant handbook. The outline content was also identified.

Details of the recommendations for an improved heat pump installation process, including an improved information leaflet, are presented in Table 4.

**Table 4.** Recommendations for improved heat pump installation service.

Service improvement	Details
An introductory letter sent to all tenants informing them of the potential change to their system, followed by a home visit to provide more detailed information	This provides an opportunity to outline the technology and its benefits to encourage uptake as early as possible. The home visit is required to explain the installation process, the system and to answer any of the tenants’ questions. The home visit also allows the landlord to obtain contextual understanding of the property, to assess the user and environment better in order to indicate any impact on the property (such as loss of space) during and after installation. The member of staff carrying out the home visits should be well informed about how the system works and what the installation process entails.
Community information event	A community information event is a beneficial approach to enable tenants to discuss with each other what they think about the systems. They should not replace home visits; the home visit is a crucial part of the process to encourage tenants to have the systems and understand the context into which it is being installed.
Information leaflet, to be left with tenants at the first home visit	Tenants need to be fully informed over the physical installation of the heat pump, the amount of space to be taken up, what changes will be made to their home and any redecoration that may be required. The tenants should also be informed about the impact to their gardens as this is a key concern for tenants and a barrier to uptake. Information about costs savings should be presented in a way that does not set too high expectations incorrectly. Any messages delivered must set appropriate expectations and provide consistent information.
Setting up the system at installation	Rather than setting the system to its default, tenants should be consulted to identify their lifestyle patterns and preferences in order to set timers and temperatures in ways that suit the tenant best but still allow the heat pump to operate effectively.
Information leaflet, with information about on-going use of the system	This includes information on how to control the basic functions of the heat pump, reducing feelings of vulnerability. It also enables tenants to operate the heating system to fit with their lifestyle, whilst still operating effectively. This reduces the demand on the landlord incurred as a result of visiting tenants for minor changes to their system.
Identification of a tenant representative(s)	A lead tenant or tenant group, a mechanism adopted in many social housing organisations, should be established to allow community discussion of the installations. This helps to provide a positive and safe route for tenants and reduces time and resource costs for the social housing staff.
Help service	Tenants should be supplied with one formal contact for any help and support, usually the social housing landlord.
On-going direct communication	Housing officers and staff who carry out visits to tenants’ properties or speak on the telephone should be knowledgeable about heat pumps so that they can communicate clearly with tenants. This might include a database including details of the heating system installed, to personalise the interaction.

The two information leaflets were developed using the requirements identified in Phase 2 and sent to social housing tenants for evaluation, alongside a survey questionnaire. Leaflet A covered how the heat pump system works, key benefits of the system and contacts for further information. This was to act as a point of reference for the tenants between introduction and installation, during the decision-making stage or post-decision, pre-installation. Leaflet B provided more detailed information about how to use the system and the controls, following the requirements set out in this paper, as well as key contact information. Cost expectations were not included as part of the written information, as the previous phase had identified that this was better done verbally to ensure tenants understood the implications regarding cost in relation to how they used the system. Factors concerning cost were mentioned in the leaflet in terms of how their operation of the heat pump affects its performance, but no definitive cost data were given. Pictures to illustrate the heat pump system were also included, to provide information about what would be installed in the home. The leaflets were checked by the social housing organisation to confirm their contents were accurate.

**Figure 3.**
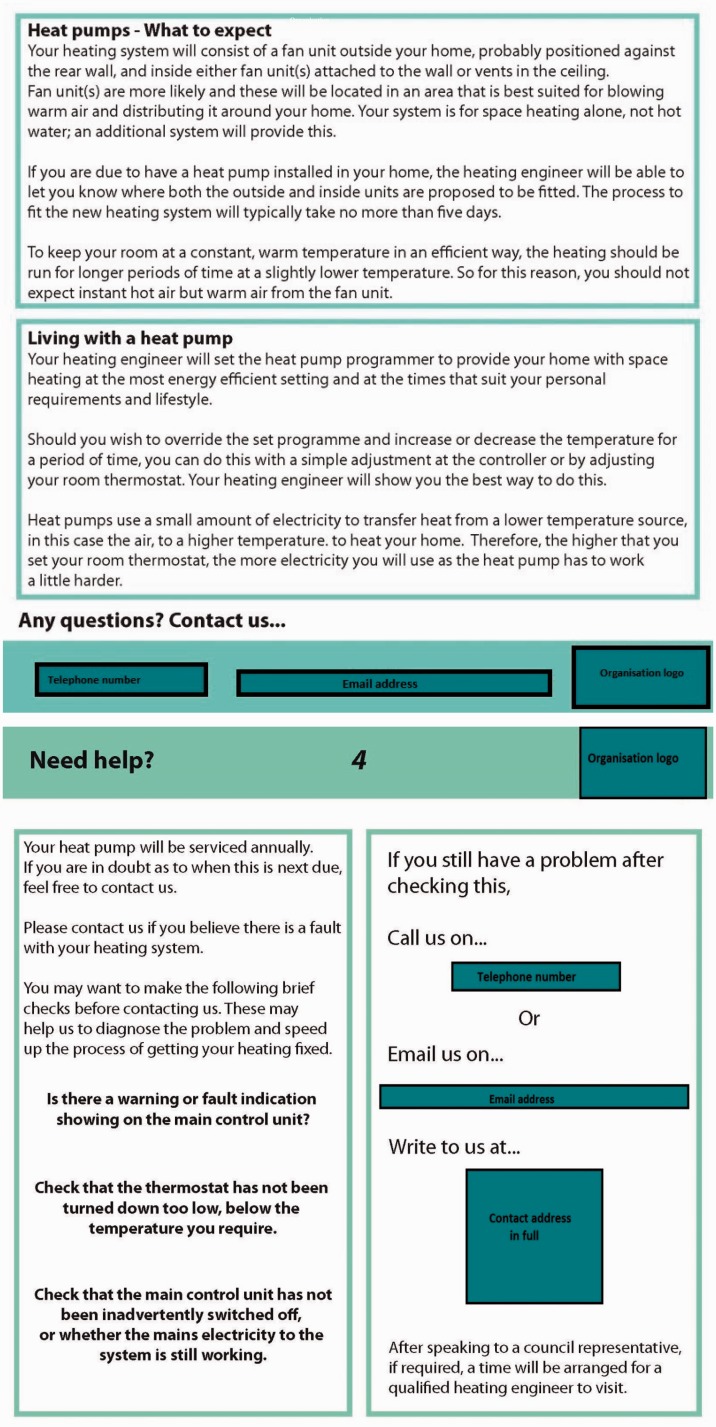
Leaflet extracts.

The length and content of the leaflets were designed with consideration to cognitive load and the impact of this on older people in particular, a significant portion of the social housing tenants. A minimum font size of 12 point was used to ensure easy reading by older readers.^29–32^ Older readers also benefit from short line lengths and left justified text^29^ and so these guidelines were followed in the design of the leaflets. Extracts of the leaflets are shown in Figure 3.

## Findings from Phase 3: Evaluation

Of 120 questionnaires distributed in the postal survey, only 15 complete responses were received from social housing tenants (response rate of 12.5%). Eight participants assessed leaflet A and seven assessed leaflet B. Responses were received from 10 female, four male and one couple; participants were from a range of ages from 25 to over 80 years. The size of the returned sample was too small to achieve any statistical significance or to be generalised to a wider population, so the data captured were explored to generate insights to inform requirements for further iterations before implementation. Overview results are reported here only where more than half of the respondents were in agreement.

Participants felt that the first impressions of the leaflet design were positive; easy to read, the right size overall and with appropriate text size. When asked about the content of the leaflet, and whether, after reading the leaflet, they felt they understood how a heat pump works, most said yes. Participants were also asked whether they felt well informed about what to expect from a heat pump installation and, again, a majority said yes. All the participants already had some experience of heat pumps and so were able to judge whether the leaflet provided them with appropriate information, based on their own expectations. Whilst it was not possible to follow up whether this understanding was accurate or not, this does suggest improvement compared with the majority of people in Phase 1 who did not feel well informed about their heat pump. Participants were also asked whether the leaflet provided adequate information about what to do if they had a query about their heat pump. The majority said they felt that the leaflet helped them find this information.

Participants who received leaflet A felt that it would have been useful to receive the leaflet at the time when they were first approached about having a heat pump. Participants who received leaflet B were asked about the information on usage of the system, through a series of scenarios where they were asked to make a judgement about whether they felt they could complete the given task using the information provided. Scenarios included changing the heating set-point and setting the timer. Participants did not feel confident in being able to do these tasks using the information provided in the leaflet. This suggests that this section still needs significant iteration before it provides clear and useful information.

## Discussion

Participants in this research referred to the installation of heat pumps as a service provided from the council or housing association to the tenants. The authority of the landlord places a particular dynamic on this service, differing, for example to a consumer service where there is a more equal dynamic. Although the tenants were referred to as ‘customers’ and the element of tenant choice was surfaced in the data, tenants indicated that they did not always feel as though they had a choice about having the new system. This was particularly evident in cases where the current heating system had broken or where it was suggested that a replacement would not be done until a later date, leading the tenant to feel as though the installation of the heat pump was inevitable. Some tenants interviewed in this research stated they felt wary about having the heat pump system installed because it was something they knew little about, and both tenants and landlords recognised this ‘fear of the unknown’ as a barrier to adoption. It is recognised that the installations were generally only possible through grants and financial schemes; social housing organisations do not have disposable funds to enable retrofit programmes of energy saving measures without subsidisation from external bodies. Consequently, the timing of heat pump installations is not wholly within the landlord’s control.

The installation of heat pumps into social housing properties involves multiple actors. The tenants are aware that multiple external organisations are involved but they experience the installation and maintenance as one service process. The tenants view the landlord as their primary service provider and they are the expected contact point for any problems. The social housing landlord, as the process facilitator, provides the service surrounding the installation from introduction of the technology to the on-going maintenance, facilitating the installation being carried out and the technology being used. Being aware of this relationship is also important to ensure the delivery of a consistent message from the various service actors working on behalf of the landlord. The research has identified that tenants received information from the installers, their landlord and, in some cases, other actors such as maintenance workers and neighbours. All social housing landlords involved in this research reported the education of tenants in understanding and using heat pump systems as the biggest and most critical issue they encountered when doing installations. Written information delivered to tenants was identified as a key touchpoint that was viewed as important from both the landlords’ and tenants’ perspectives. Aside from written materials, verbal communication played an important part in educating tenants; therefore, it is important that this message is consistent and accurate.

The findings of this research have identified that social housing tenants can lack confidence when using their heat pump system controls. Tenants often reported that they did not want to touch the controls as they felt they could not operate them, exacerbated by a lack of appropriate information provided. In many cases, tenants were remaining dependent on the landlord to operate controls for them. Given the importance of a heating system in a tenant’s daily life, this can cause significant problems.

Standardisation of control panels across the different types of heat pump systems emerged as a potential avenue for improvement of heat pump systems so that landlords could choose a supplier who offered such products; this would become a market differentiator for the heat pump manufacturer. The importance for all actors who interacted with tenants to have detailed and up to date knowledge on heat pump use and energy tariffs was also identified as a critical part of the service provision.

Some tenants reported dissatisfaction with the heat pumps because they did not produce the expected heat. This was often when the heat pump was not fully understood by the tenant, who expected, but did not experience, noticeable radiant heat from their heat emitters. Improved information to all tenants with a heat pump will help manage expectations as well as identify how tenants can use the system efficiently whilst achieving satisfactory thermal comfort. The desire to be able to alter the temperature to suit residents’ individual needs is one of the key drivers for tenants to have control over their systems. Some social housing landlords reported that heat pumps are installed with default settings; they then work with tenants to adjust the systems to their preferences. Whilst this is a positive step towards ensuring individual needs are catered for, some information, such as relevant health issues or patterns of occupancy, could be obtained in advance to better determine a bespoke set up. However, this is an area that still requires significant research, to ensure personalised settings can also be energy and cost efficient. The lack of optimisation can have a significant impact on energy use and subsequent carbon emissions if secondary heating or ventilation is used as a workaround to the problem.

### Applying a user centred design approach to a service

Through the evidence of the studies conducted, insights gathered, greater understanding obtained and resulting recommendations, it can be seen that a user centred approach can be applied to the service of installing heat pumps into social housing and deliver valuable information with a view to improving the service provided. The involvement of representative of both service recipients and service providers helped to understand the context and requirements of both sides of the service. This allowed for an holistic view of the service and identification of where the positive and negative aspects lay in its delivery. This ensured that the service offering, in this case a heat pump, was considered within a realistic context, identifying appropriate communication touchpoints between the keys actors. This enabled the management of expectations of the service delivery, through clearer communication of the use and performance of a heat pump. This approach could equally apply to other new energy technologies in social housing where landlords may not have a good awareness of the context in which the technology will be used and tenants may not fully understand the purpose and optimum use of the technology.

The initial exploration of the context of use is the stage where the user centred design process is most noticeably impacted by the complexity of a service. The overall service delivery may be hindered by multiple interactions, or touchpoints, which make up the service, which was confirmed by this research in social housing, where there is an on-going relationship between tenants and landlords, something that is not present with owner–occupiers. Additionally, the holistic review of the service presented opportunities for multiple new aspects to improve the service. However, the scope of all these factors is too wide to deal with in just one user centred design process cycle. Therefore, the user centred design process when applied to a service is likely to involve a greater level of iteration, to consider the overall service delivery and the touchpoints which form the service. It is key to note here, however, the value in applying user centred design to the holistic service and not just to the individual touchpoints. Firstly, the in-depth review with service recipients and service providers of the holistic service allowed identification of the touchpoints that needed addressing, improving or creating, that would not have been realised prior to the involvement of users. Secondly, improvements to the touchpoints could be designed within the service context. Other aspects of the service may interact with or impact on a touchpoint and were considered in its development. If touchpoints had been explored in isolation, even through a user centred process, they would be unlikely to be successful when experienced in the service as a whole.

Ideally, actors within the service delivery should be involved in wider aspects of design improvement, including identification of the need for, and development of, the technologies from the outset. This may include ascertaining the specific requirements for heating systems, the design of intuitive controls, the development of intelligent feedback systems and the provision of appropriate communication tools to provide comfortable, efficient and cost-effective living environments. When user centred design is included in the complete design process (from the ‘fuzzy front end’^33^ through to evaluation post-installation), solutions that truly meet all users’ needs can be created.

## Limitations of the research

This research relied on the involvement of social housing landlords and tenants as participants in the various studies. This is a key attribute of user centred design, but encouraging participation can be difficult. Whilst the social housing organisations involved in the three phases were enthusiastic and helpful, sample sizes were sometimes small, particularly in Phase 3: Evaluation. The response rate in this phase was reasonable at 12.5%, but the study relied on the social housing landlords to distribute the leaflets and questionnaires, due to data protection limitations; the researchers were not able to contact potential participants directly. The number of potential participants was also limited, as the questionnaire could only be sent to households who had a heat pump; 120 in total. The involvement of just one social housing provider at this stage also limited the population from which to draw the sample, but inclusion of other social housing organisations would have required the development of bespoke leaflets for each provider, beyond the scope of the research. However, the principle of the user centred design approach is demonstrated through the phases described in the paper and the inclusion of studies even with small samples shows how the stages of user centred design can be applied in practice. Further research with a wider sample would clearly provide more reliable data and improved iterative design of the intervention leaflets.

## Conclusion

In this research, a user centred design process was followed to explore the context of heat pump use in social housing, refine and develop user requirements for an improved service delivery of heat pumps in social housing and design then evaluate one particular part of that service, an information leaflet. The service was considered primarily from the perspective of two main actors: the social housing landlords and tenants. The use of this methodology enabled in-depth exploration of the process involved in heat pump installations in social housing. Through this, processes across different social housing organisations were reviewed and people’s experiences investigated to establish a substantial, contextual understanding. Users were involved in all phases of the research, from exploration through to evaluation, as is the underlying fundamental principle of user centred design.

This deep and rich exploration, with both the service recipients and service providers, enabled tenant requirements to be identified, defined and then balanced against the opportunities and restrictions of the service delivery highlighted by the service providers. This understanding of both sides of the service process enabled the identification of where the introduction, installation and on-going use of the heat pump system could be improved.

Consistent requirements have been identified for improved information to be delivered to tenants both verbally and in written format, at the point of introducing the technology and when the heat pump is installed, to fully inform the tenant about what to expect from their heat pump and how to use it. User-friendly reference materials for how to use the system controls have been outlined in the paper, to replace the overly-complicated technical manual aimed at installers. This reduces feelings of frustration and vulnerability of tenants through reducing the dependency on others to operate their domestic heating. Improved understanding of the heat pump also enables the system to be used efficiently, avoiding wasted heat and unnecessary carbon emissions.

Applying user centred design to the service delivery of installing heat pump systems into social housing is a new way of approaching the design of this service process. In a relatively immature area of industry, installations to date have been heavily focused on the technical aspects of the system. There is an increasing recognition of the importance of the end user, their experiences and behaviours. To increase successful integration of renewable technology into the UK domestic sector, people need to accept and adopt the technology and use it to best effect. This places significant importance on understanding the dwellings occupants and the service provision to achieve this successful adoption and use of renewable technology. There is clearly a need for further research in this area, to better understand at scale how technologies such as heat pumps are being used in social housing and whether they are actually saving energy, whilst delivering good thermal comfort. Social housing organisations are encouraged to apply a user centred design approach to their service provision, to ensure that they are providing a service that is meeting the needs of *all* the actors.

## Authors’ note

The full thesis on which this paper is based is available from the Loughborough University Institutional Repository (https://dspace.lboro.ac.uk/2134/14485). Access to the underlying research data presented in this paper is restricted as participants may be identifiable.
